# Baicalein inhibits prostate cancer cell growth and metastasis via the caveolin-1/AKT/mTOR pathway

**DOI:** 10.1007/s11010-015-2429-8

**Published:** 2015-05-10

**Authors:** Zhaoxin Guo, Xiaolin Hu, Zhaoquan Xing, Rui Xing, Renguang Lv, Xiangyu Cheng, Jing Su, Zunlin Zhou, Zhonghua Xu, Sten Nilsson, Zhaoxu Liu

**Affiliations:** Department of Urology, Qilu Hospital of Shandong University, Jinan, 250012 Shandong China; Department of Endocrinology, Jinan Central Hospital, Shandong University, Jinan, 250013 Shandong China; Department of Urology, Linyi County People’s Hospital, Dezhou, 251500 Shandong China; Department of Urology, Jinan Iron and Steel General Hospital, Jinan, 250000 Shandong China; School of Nursing, Shandong University, No. 107 Wenhua Xi Road, Jinan, 250012 Shandong China; Department of Oncology and Pathology, Karolinska Institutet, 17177 Stockholm, Sweden

**Keywords:** Baicalein, Anti-cancer activity, Androgen-independent prostate cancer, Metastasis, Caveolin-1

## Abstract

Prostate cancer (PCa) is lethal type of genitourinary cancer due to its high morbidity and gradual resistance to androgen deprivation therapy. Accumulating evidence has recently suggested that the daily intake of flavonoids is negatively correlated with the risk of cancer. In this study, we aimed to investigate the potential effects of baicalein on androgen-independent PCa cells and the underlying mechanisms through which baicalein exerts its actions. Cell viability and flow cytometric apoptosis assays indicated that baicalein potently suppressed the growth and induced the apoptosis of DU145 and PC-3 cells in a time- and dose-dependent manner. Consistently, the inhibitory effects of baicalein on migration and invasion were also observed in vitro. Mechanistically, we found that baicalein can suppress caveolin-1 and the phosphorylation of AKT and mTOR in a time- and dose-dependent manner. Moreover, the inhibition of the activation of AKT with LY294002 significantly promoted the apoptosis and metastasis induced by baicalein. In conclusion, these findings suggested that baicalein can induce apoptosis and inhibit metastasis of androgen-independent PCa cells through inhibition of the caveolin-1/AKT/mTOR pathway, which implies that baicalein may be a potential therapeutic agent for the treatment of androgen-independent prostate cancer patients.

## Introduction

Prostate cancer (PCa) is the most common malignant cancer in men and the second leading cause of cancer-related death being only inferior to lung cancer [1]. At present, androgen deprivation therapy (ADT) is still the main treatment for PCa. However, within 2 years, the majority of prostate cancer becomes androgen independent and progresses into castration-resistant prostate cancer (CRPC) stage, at which point few drugs are effective, giving rise to high mortality [2, 3]. Therefore, the development of pharmacological interventions to retard prostate tumor growth after the emergence of androgen-independent disease is urgently needed.

Flavonoids are widely used Chinese herbal medicines. Epidemiological studies now indicate that an increased intake of dietary flavonoids is associated with a decreased risk of some cancers [4, 5]. Baicalein is a bioactive flavonoid derived from the root of *Scutellaria baicalensis*, and has been widely used to treat inflammation, cardiovascular diseases, and infections [6]. Baicalein alone, or in combination with other agents, can also inhibit cancer cell growth and metastasis in breast cancer, hepatocellular carcinoma, leukemia, and colon cancer [7–10]. In addition, Chen previously found that baicalein can regulate the expression of the androgen receptor, inhibit the proliferation of prostate cancer cells, and induce the apoptosis of these cells [11]. However, the effects of baicalein on the metastasis of androgen-independent prostate cancer cells and the underlying molecular mechanisms have not been well established.

In this study, the DU145 and PC-3 cell lines were selected as androgen-independent prostate cancer cells. Our data established the growth-inhibitory and pro-apoptotic effects of baicalein on PCa cells in vitro. Simultaneously, we found that baicalein can potently inhibit the migration and invasion of PCa cells. Further investigation of the underlying mechanisms indicated that the caveolin-1/AKT/mTOR pathway plays key roles in these actions. The effects of baicalein on androgen-independent cells provide useful information for the treatment of CRPC.

## Materials and methods

### Cell lines and reagent**s**

This study was approved by the Qilu Hospital Committee of Shan Dong University. Androgen-independent PCa cell lines (DU145 and PC-3) were purchased from the Type Culture Collection of the Chinese Academy of Sciences (Shanghai, China). All of the cells were grown in RPMI-1640 medium (Hyclone, Utah, USA) containing 10 % fetal calf serum (Gibco, CA, USA) at 37 °C in a 5 % CO_2_ incubator. Baicalein was purchased from Selleck Chemicals (Houston, TX, USA) and was dissolved according to the manufacturer’ instructions. Antibodies against phospho-AKT (Ser473), AKT, phospho-mTOR, mTOR and GAPDH, and HRP-conjugated goat anti-rabbit and anti-mouse IgG antibodies were obtained from Cell Signaling Technology (Beverly, MA, USA). Antibodies against cleaved PARP, Bax, Bcl-2, and survivin were obtained from Abcam (Abcam, Cambridge, UK).

### Cell viability assay

The cell viability was determined by the CCK8 assay. Briefly, DU145 or PC-3 (4 × 10^3^ cells/well) cells in 200 μl of medium were seeded into 96-well plates. After 12 h of incubation to allow adherence, the medium was replaced with medium containing different concentrations of baicalein and the plate was incubated for 24, 48, and 72 h. The cultured cells were subsequently treated with 20 μl of Cell Counting kit-8 (Dojindo, Japan) and incubated at 37 °C for an additional 4 h according to the manufacturer’s instructions. The absorbance values were determined at 450 nm using a microplate reader (Bio-Rad, Hercules, CA, USA).

### In vitro scratch assay

The PCa cells were seeded in 24-well plates. After incubation for 24 h, each well was manually scratched with a 200 μl pipette tip, washed three times with PBS, and incubated with baicalein (20 and 40 μM, respectively) at 37 °C. Images were obtained again after 24 h of incubation. The distance between two cell edges was analyzed using the ImageJ software.

### Transwell invasion and migration assay

The Transwell invasion assay was performed as described previously [12]. Briefly, a total of 4 × 10^4^ cells suspended in 100 μl of serum-free medium were added to the upper chambers of the Transwell system (24 wells, 8-mm pore size; Corning Costar, Lowell, MA, USA) coated with 2 mg/ml Matrigel (BD Biosciences). RPMI-1640 containing 20 % FBS and baicalein (20 and 40 μM) was then added to the lower chamber. After 24 h, the non-invaded cells in the upper chamber were gently removed with a cotton swab, and the cells attached to the lower surface were fixed with precooled methanol and stained with 0.1 % crystal violet. Five fields of each chamber were randomly selected, and the cell numbers were counted under a microscope.

For the migration assay, the cells were seeded into upper chambers that were not coated with Matrigel. The following steps in the assay were the same in the invasion assay. After 24 h, the cells on five randomly selected fields in the lower surface were counted, and the cell numbers were then subjected to statistical analysis.

### Apoptosis assay

Cell apoptosis was detected using the Annexin V-FITC/PI apoptosis detection kit (BD Biosciences, San Jose, CA, USA). In brief, 1 × 10^5^ untreated or baicalein-treated cells were harvested following trypsinization and centrifugation. The cells were washed with PBS and resuspended in 500 μl of binding buffer. After 5 μl of Annexin V-FITC was added, the cells were incubated after 5 min 5 μl of PI (propidium iodide) was added, and the cells were incubated 15 min in the dark. The data were analyzed using the WinMDI V2.9 software (The Scripps Research Institute, San Diego, CA, USA).

### Western blotting

The cells were lysed in RIPA buffer containing 1 % protease inhibitors. Equal amounts of proteins from each sample were separated by SDS-PAGE and transferred onto a PVDF membrane using a wet transfer apparatus (Bio-Rad, Hercules, CA, USA). The membranes were blocked with 5 % non-fat milk for 2 h at room temperature and incubated with the primary antibodies overnight at 4 °C. The membranes were then exposed to the horseradish peroxidase-labeled secondary antibodies for 1 h at room temperature and detected using an enhanced chemiluminescence detection system (Amersham, Pittsburg, PA, USA). The protein levels were analyzed using the ImageJ software.

### Statistical analysis

The data are mostly presented as the means ± SD. The SPSS software package (version 13.0; SPSS, Chicago, IL, USA) was used for all of the statistical analyses. Significant differences between the treatment and control values were analyzed by Student’s two-tailed *t* test or one-way analysis of variance wherever appropriate. Differences were considered statistically significant if *P* < 0.05. Each variable was tested twice and the experiments were repeated three times.

## Results

### Baicalein inhibits proliferation of prostate cancer cells

**Fig. 1:**
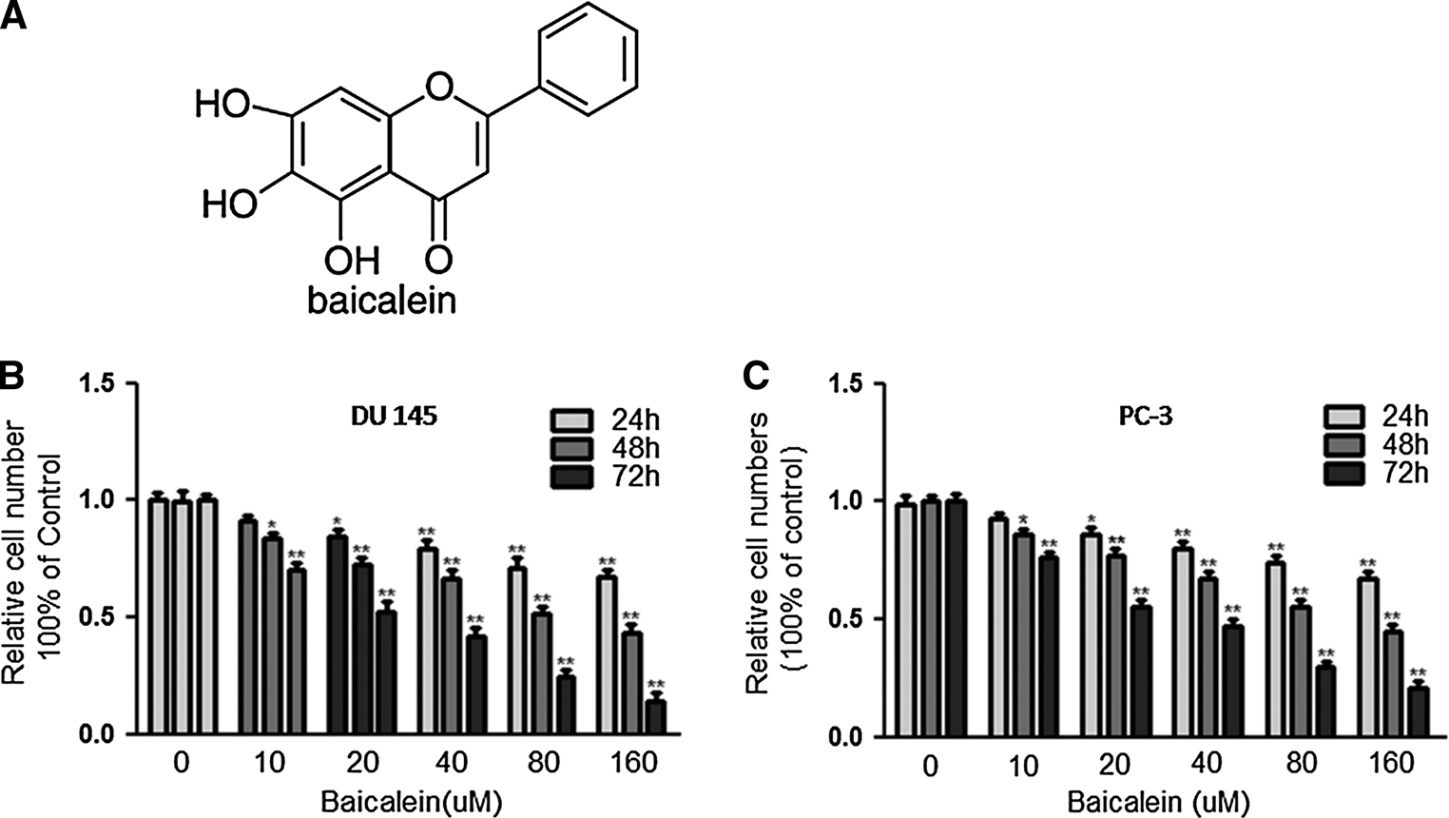
Baicalein suppresses the viability of DU 145 and PC-3 cells. **a** Structure of baicalein. The effect of baicalein on cell viability was measured by the CCK-8 assay. **b** DU145 cells and **c** PC-3 cells were treated with different concentrations of baicalein for 24, 48, and 72 h. Baicalein significantly inhibited the viability of both cell lines in a dose-and time-dependent manner. The results are presented as the means ± SD of three independent experiments and the corresponding standard error. *P < 0.05; **P < 0.01

To study the effects of baicalein on the proliferation of PCa cells, DU145 and PC-3 cells were exposed to different concentrations of baicalein for 24, 48, and 72 h, and their proliferation was analyzed using the CCK-8 assay. The results showed that baicalein significantly inhibited the proliferation of DU145 and PC-3 cells in a time- and dose-dependent manner (*P < 0.05, **P < 0.01) (Fig. 1b, c). The viability of DU145 and PC-3 cells was reduced to 58.4 and 52.7 % after treatment with baicalein (40 μM) for 72 h. In addition, the IC50 values of baicalein in DU145 and PC-3 cells were between 20 and 40 μmol/l. We then selected the baicalein concentrations of 20 and 40 μmol/l for the subsequent experiments.

### Baicalein induces apoptosis in prostate cancer cells

**Fig. 2:**
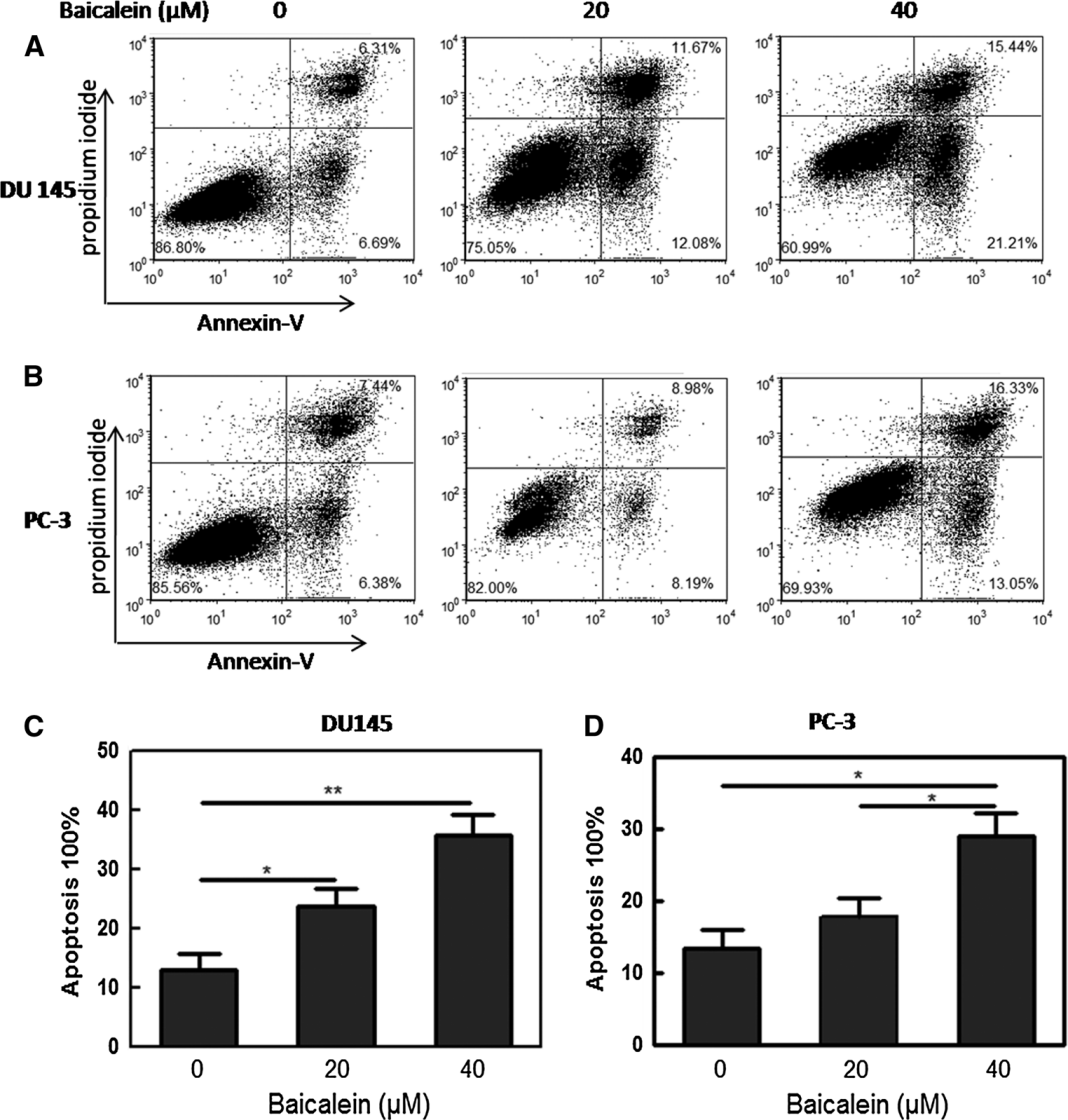
Baicalein induces dose-dependent apoptosis in DU145 and PC-3 cells. **a** DU145 and **b** PC-3 cells were treated with baicalein (0, 20, and 40 μM) for 24 h and stained with FITC-Annexin V and PI. The percentage of surviving cells is shown in the *lower left* quadrant; and the percentages of early-stage and late-stage apoptotic cells are shown in the *lower right* and *upper right* quadrants, respectively. **c**, **d** The apoptosis induced by baicalein was quantified. The *Data* are presented as the means ± SD of three independent experiments. *P < 0.05; **P < 0.01

To investigate the effect of baicalein on apoptosis in PCa cells, staining with Annexin V-conjugated Alexa Fluor 488 and propidium iodide was used to analyze the percentage of apoptotic cells induced by baicalein. The lower right (LR) and upper right (UR) quadrants of the histograms showed the percentages of early and late apoptotic cells, respectively (Fig. 2a, b). The total percentage of apoptotic cells (UR + LR) increased from 13 % in control DU145 cells to 23.75 and 36.65 % in the cells treated with baicalein (20 and 40 μM, respectively) for 48 h (*P < 0.05, **P < 0.01) (Fig. 2c). A similar phenomenon was also found in the PC-3 cells, and the total percentage of apoptotic cells was increased from 13.82 to 17.17 % and 29.38 %, respectively (*P < 0.05, **P < 0.01) (Fig. 2d). The treatment of DU145 and PC-3 cells with 20 and 40 μM baicalein for 48 h induced apoptosis in both cell lines in a dose-dependent manner, indicating the anti-tumor effect of baicalein on PCa cells.

### Migration and invasion are inhibited by baicalein in prostate cancer cells

**Fig. 3:**
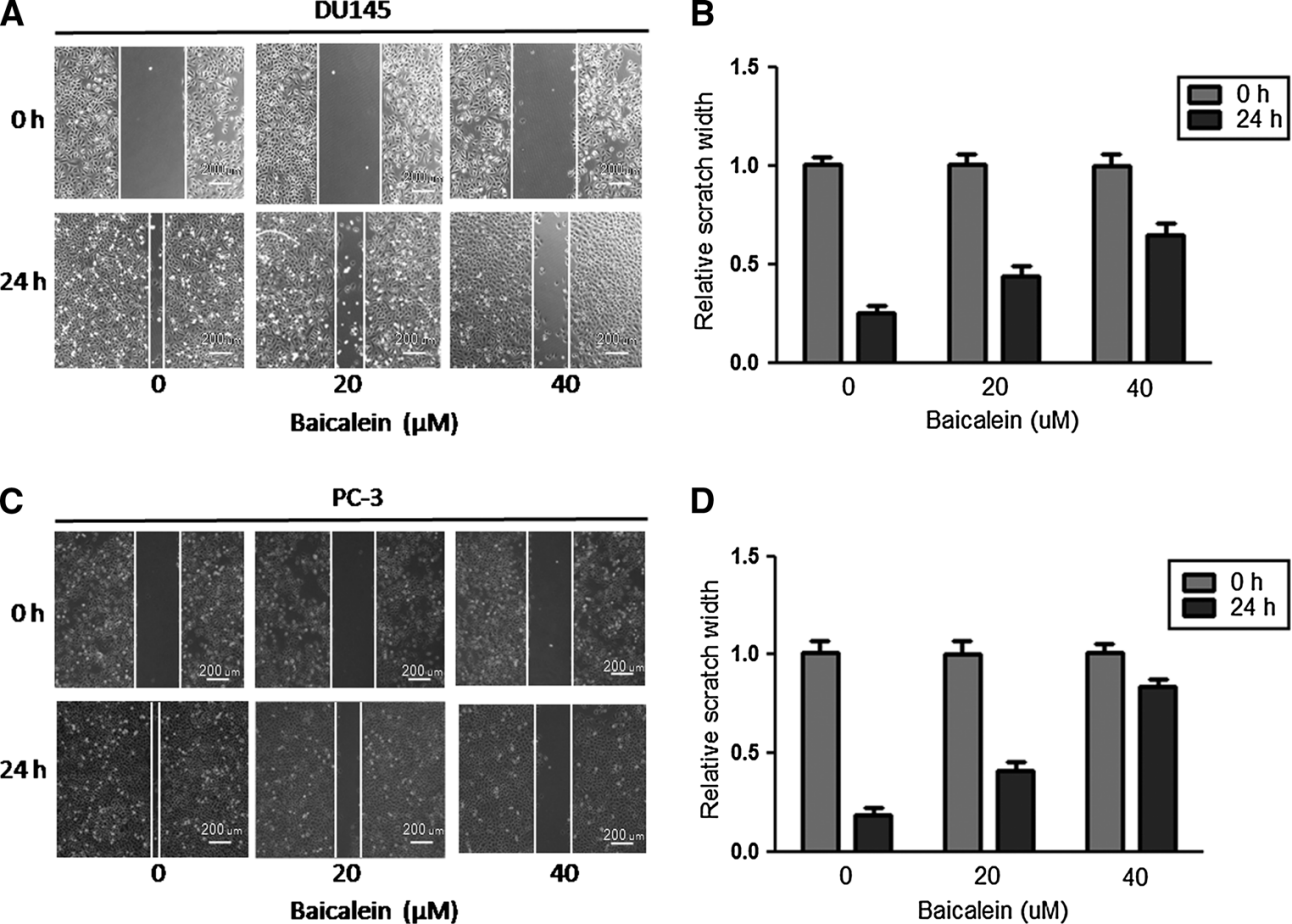
The scratch assay shows the effect of baicalein on cell migration. **a** Scratch assay of DU145 cells treated with 0, 20, and 40 μM baicalein. **b** The inhibition of migration was transformed to the percentage of the initial distance between the two edges. The baicalein-treated DU145 cells showed a lower rate of wound closure than the control cells. **c** Scratch assay of PC-3 cells treated with 0, 20, and 40 μM baicalein. **d** The baicalein-treated PC-3 cells showed a lower rate of wound closure than the control cells

**Fig. 4:**
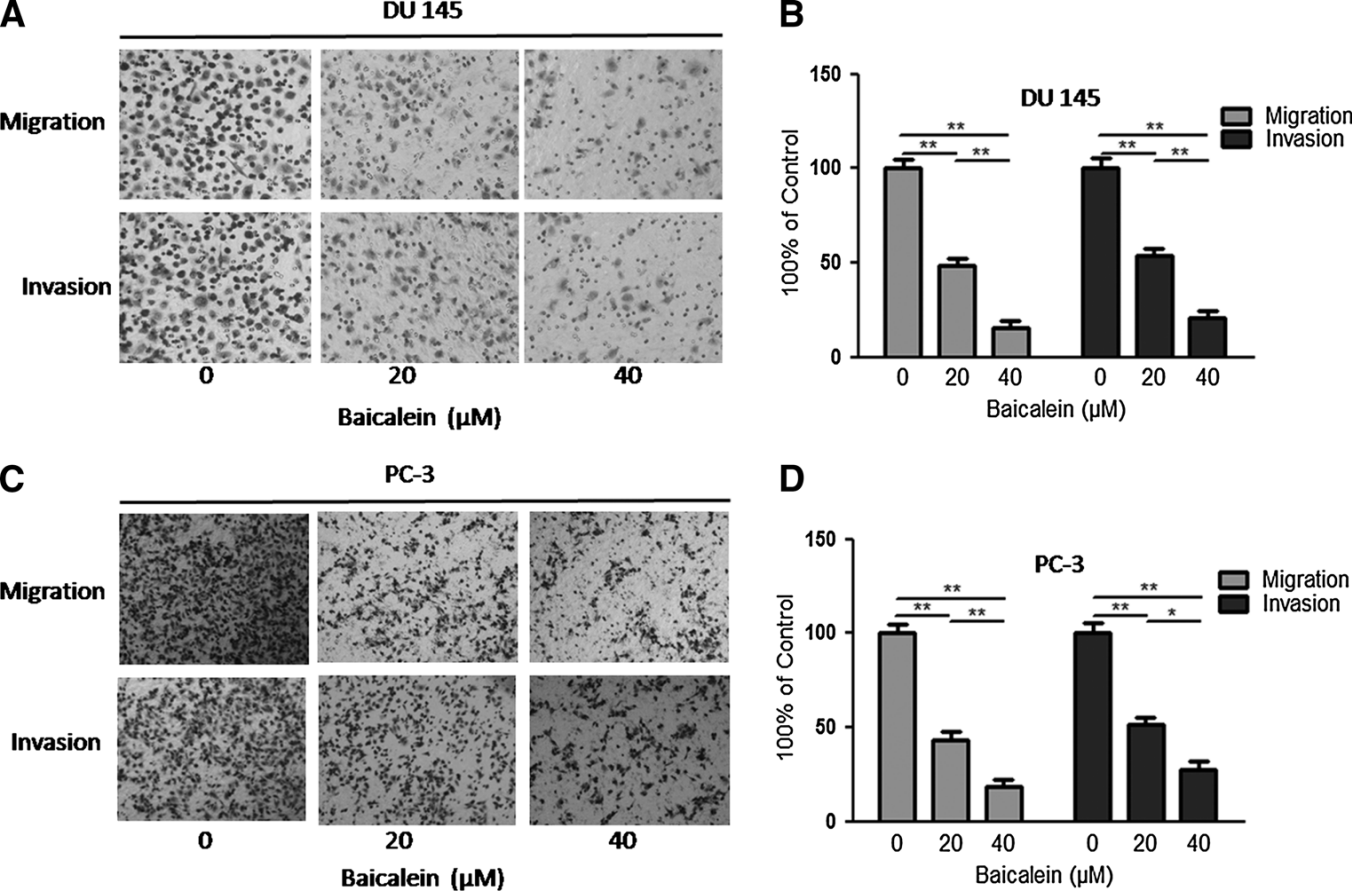
Baicalein inhibits cell migration and invasion ability in a dose-dependent manner. **a** The migration and invasion of DU145 cells were inhibited after treatment with different concentrations of baicalein. **b** The number of DU145 cells that successfully migrated and invaded was counted. **c** Treatment with 0, 20, and 40 μM baicalein inhibited the migration and invasion of PC-3 cells. **d** The decrease in the number of PC-3 cells indicates the marked inhibitory effect of baicalein on cell mobility. The *Data* are presented as the means ± SD of three independent experiments. *P < 0.05; **P < 0.01

The scratch assay was implemented to investigate the effect of baicalein on the migration of prostate cancer cells. As shown in Fig. 3a, b, the migration of DU145 cells was restrained by baicalein in a dose-dependent manner, and a similar effect was also observed in PC-3 cells (Fig. 3c, d). A transwell assay was then performed to further test the influence of baicalein on cell migration and invasion. Our results showed that baicalein can significantly inhibit DU145 cells migration and invasion (*P < 0.05, **P < 0.01) (Fig. 4a, b) in a dose-dependent manner. Furthermore, we observed the same potent effect of baicalein on PC-3 cells (*P < 0.05, **P < 0.01) (Fig. 4c, d).

### Effects of baicalein on the expression of cell apoptosis-related proteins

**Fig. 5:**
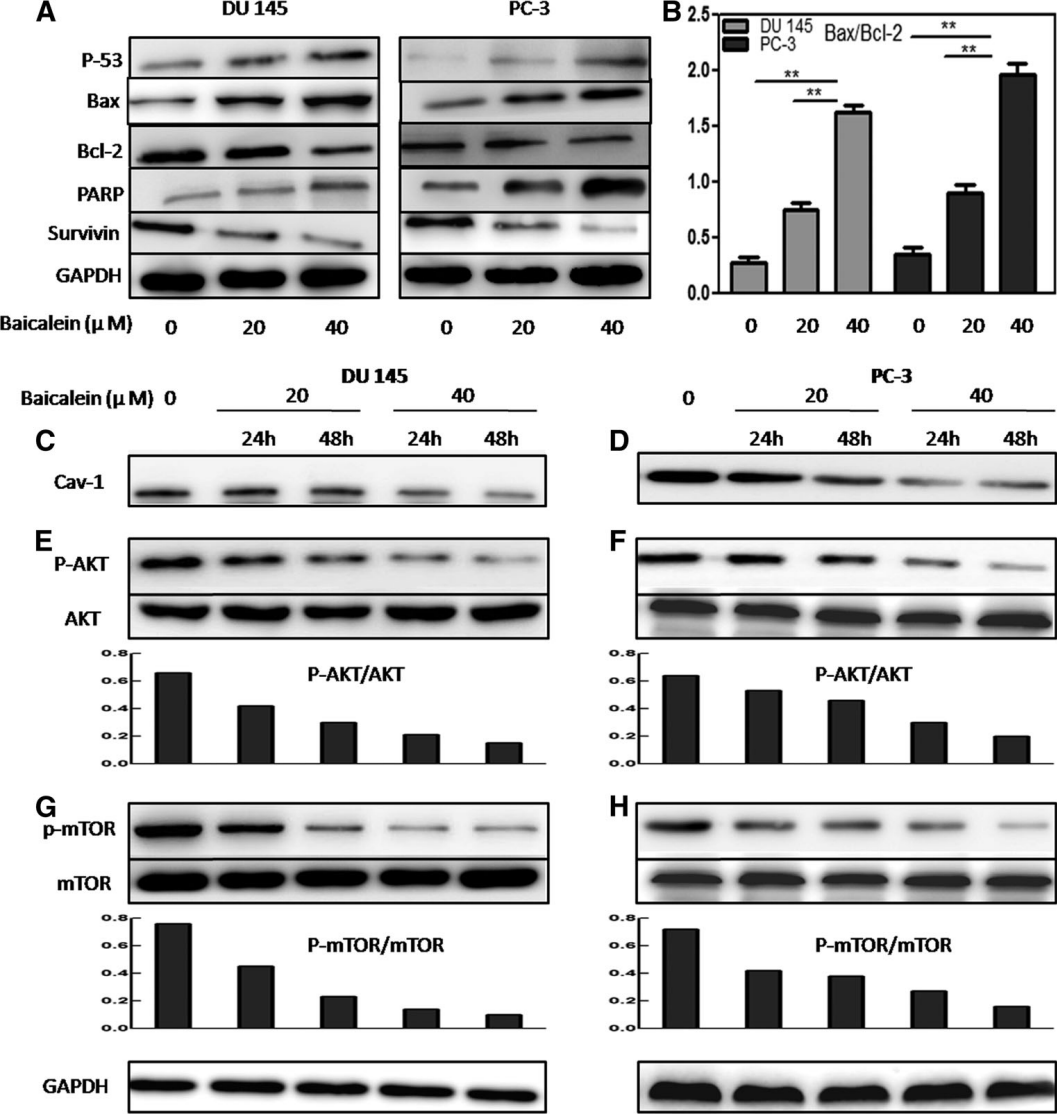
Effects of baicalein on the protein levels of Bax, Bcl-2, PARP, survivin, caveolin-1, AKT, and mTOR in DU145 and PC-3 cells. **a** Bax, Bcl-2, PARP, and survivin were assayed in DU145 and PC-3 cells by Western blotting analysis using GAPDH as a control. **b** Change in the Bax/Bcl-2 ratios in DU145 and PC-3 cells after treatment with baicalein. The densitometry value of each band was determined with ImageJ. The Data are presented as the means ± SD of three independent experiments. *P < 0.05; **P < 0.01. **c**–**g** The levels of caveolin-1, AKT, p-AKT, mTOR, and p-mTOR were analyzed by Western blotting. The p-AKT/AKT and p-mTOR/mTOR ratios were quantified by densitometry analysis

It is well known that the expression of the pro-apoptotic protein Bax is associated with the increased apoptosis, whereas the anti-apoptotic protein Bcl-2 is associated with the inhibition of apoptosis in target cells [13, 14]. In addition, the Bax/Bcl-2 ratio plays a role in the regulation of apoptosis. As shown in Fig. 5a, the level of Bax was elevated, whereas the level of Bcl-2 was decreased after treatment with baicalein. The comparison of the intensity of their bands revealed that the Bax/Bcl-2 ratio was increased after baicalein treatment in a dose-dependent manner (Fig. 5b). Additionally, the expression of survivin was decreased, whereas the level of cleaved PARP was elevated.

### Baicalein inhibits the proliferation and metastasis of PCa cells via the caveolin-1/AKT/mTOR pathway

A Western blotting assay was performed to detect the underlying mechanism through which baicalein exerts its actions. Caveolin-1 (cav-1) has been reported to mediate survival and promote metastatic activities in prostate cancer cells [15]. We found that treatment with baicalein resulted in a dose- and time-dependent decrease in cav-1 expression in PCa cells (Fig. 5c, d). Previous studies have indicated that the PI3K/Akt/mTOR axis plays important roles in cancer cell proliferation, metabolism, migration, and angiogenesis [16–18], and that the inhibition of the AKT signaling pathway can induce apoptosis and prevent the invasion and metastasis of prostate cancer cells. Therefore, we tested whether baicalein affects the PI3 K/Akt/mTOR pathway. The treatment of DU145 and PC-3 cells with baicalein (20 and 40 μM) for 24 and 48 h resulted in a marked downregulation of the phosphorylation levels of AKT and mTOR. In addition, the p-mTOR/mTOR and p-AKT/AKT ratios were also decreased in a dose- and time-dependent manner (Fig. 5e, f, g, h).

**Fig. 6:**
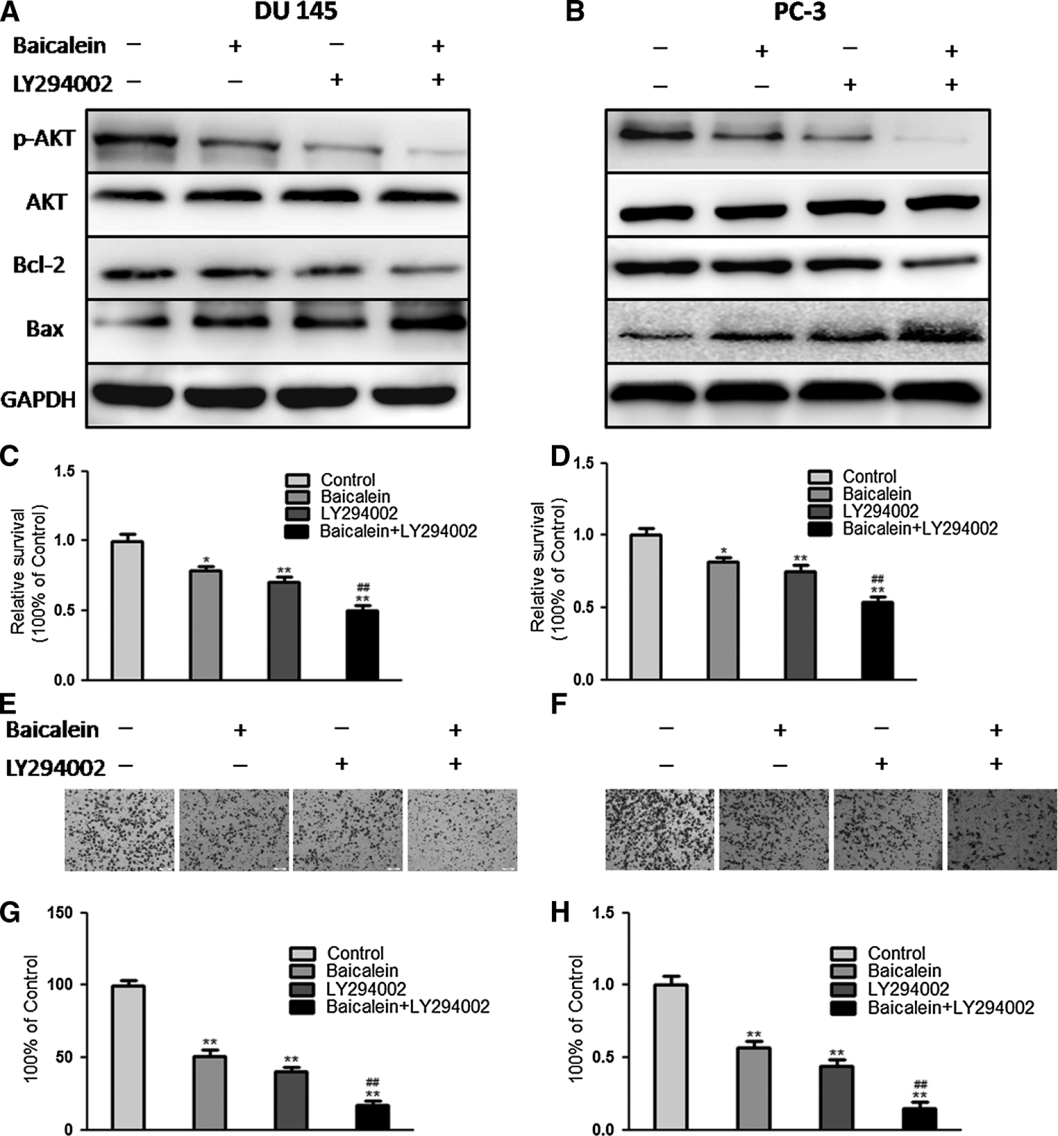
Inhibition of AKT activation promotes apoptosis and inhibits the metastasis of PCa cells induced by baicalein. **a**, **b** Expression of p-AKT, AKT, Bax, and Bcl-2 in DU145 and PC-3 cells after treatment with baicalein and/or LY294002. After treatment with LY294002A, DU145 and PC-3 cells were incubated in the absence or presence of baicalein (40 μM) for 48 h. The cell viability was measured by MTT assay (**c**, **d**), and the cell invasion was measured by a Transwell assay (**e**, **f**). The results are presented as the means ± SD of three independent experiments. *P < 0.05; **P < 0.01, compared with the control cells. ^#^ *P* < 0.05 or ^##^ *P* < 0.01, compared with the cells treated with baicalein

**Fig. 7:**
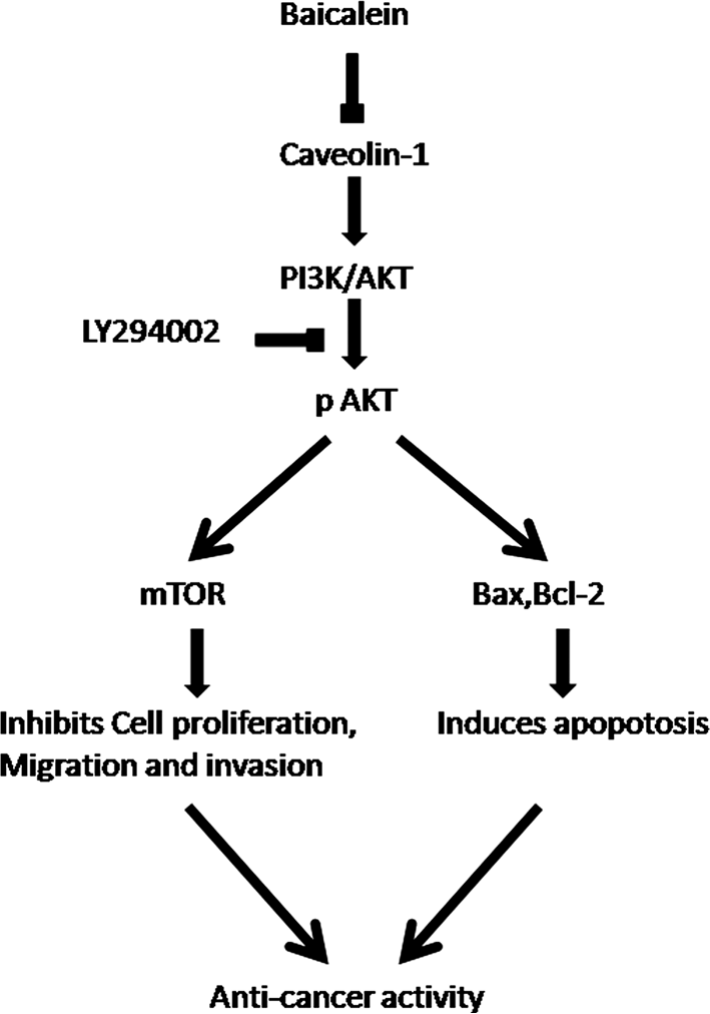
Hypothesized model for the mechanism through which baicalein induces apoptosis and inhibits metastasis of prostate cancer cells. Exogenous baicalein promotes the apoptosis and inhibits the metastasis of androgen-independent prostate cancer by interrupting the caveolin-1/Akt/mTOR pathway

To investigate the role of AKT in the proliferation and mobility of PCa cells, DU145 and PC-3 cells were pretreated with a pan-PI3 K inhibitor (LY294002, 50 μM) for 30 min and then incubated in the presence or absence of baicalein (20 μM) for 48 h. The Western blotting analysis results indicated that the inhibition of AKT activity with LY294002 could enhance the upregulation of Bax expression and downregulation of Bcl-2 expression induced by baicalein (Fig. 6a, b). We then evaluated the baicalein-induced apoptosis and anti-metastasis activity in PCa cells pretreated with LY294002. Consistently, the results showed that LY294002 significantly induced apoptosis (Fig. 6c, d) and suppressed cell invasion (Fig. 6e, f), indicating that AKT plays important roles in the apoptosis and invasion of PCa cells. In addition, the results revealed that LY294002 can markedly potentiate the pro-apoptotic and anti-metastasis effects of baicalein, which suggests that blocking the AKT signaling pathway may enhance the anti-cancer effect of baicalein (Fig. 7).

## Discussions

Flavonoids are a subclass of polyphenolic compounds that have been widely used for thousands of years in Oriental medicine due to their anti-allergic, anti-inflammatory, and anti-microbial effects [19–21]. Recently, a series of studies have shown that flavonoids exhibit anti-tumor effects against various cancer cells of different origins [7, 22, 23]. The widespread distribution of flavonoids, their variety, and their relatively low toxicity compared with other active plant metabolites (e.g., alkaloids) had led to their consumption by human beings in significant quantities [24]. Although low water solubility, fast oxidative degradation, and fast metabolism limit its pharmaceutical use in some degree, various methods have been used to overcome these issues of flavonoids [25]. The flavonoids family consists of various members, including flavones, flavonols, baicalensis, and anthocyanidins [26]. Of these drugs, the flavonoid baicalein has been found to promote the apoptosis of prostate cancer cells by regulating AR [27]. However, the effect of baicalein on apoptosis-related proteins in PCa cells and the effect of baicalein on the metastasis of PCa cells have not been elucidated.

In the present study, we demonstrated that baicalein can significantly inhibit the proliferation and induce apoptosis of androgen-independent prostate cancer cells, in accordance with previous research findings [11]. We further explored the molecular mechanisms through which baicalein induces apoptosis. The results indicated that the regulation of cav-1 and apoptotic proteins is required. Our data indicate a new mechanism through which baicalein exhibits its pro-apoptotic effect in prostate cancer cells.

Cav-1 is a major structural component of the caveolae, which are specialized plasma membrane invaginations involved in molecular transport, cell adhesion, and signal transduction [28]. Cav-1 was recently found to function as an anti-apoptotic protein, which was associated with several members of the Bcl-2 family of proteins [29–31]. In the present study, we found that baicalein reduces a time- and dose-dependent inhibition of cav-1, as demonstrated by a Western blot assay. Additionally, after exposure to different concentrations of baicalein, the pro-apoptotic Bax and PARP in DU145 and PC-3 cells were up-regulated, whereas the anti-apoptotic Bcl-2 and survivin were down-regulated in a dose-dependent manner.

The presence of metastasis is the main cause of mortality in patients with prostate cancer. Metastasis is a complex multistep process involving cell motility and invasion. Hence, the interruption of these steps is one approach for anti-metastatic therapy [32]. As shown through wound healing and Matrigel chamber invasion assays, baicalein markedly inhibited the migration and invasion of PCa cells in a concentration-dependent manner. Further research of the molecular mechanisms indicated that the cav-1/AKT/mTOR signal pathway plays a vital role in this process.

With the exception of its effect on cell apoptosis, cav-1 was also reported to be associated with poor prognosis and metastasis in prostate cancer, and a reduction in cav-1 expression can decrease the tumorigenic and metastatic potential of prostate cancer, indicating that cav-1 is a potential target for prostate cancer [33–35]. PI3K/AKT is involved in a number of important cellular processes, including cellular survival and tumor metastasis [36]. Recent studies have indicated that the PI3K/Akt signaling pathway is overactive in a large range of cancers, and the inhibition of this pathway is considered a novel target for cancer therapy [37, 38]. Moreover, it has been reported that PI3K/Akt exists in the caveola and is regulated by cav-1 [39, 40]. In our study, we found that baicalein can significantly suppress the phosphorylation/activation of AKT in a time- and dose-dependent manner, in accordance with the inhibition of cav-1. To further investigate whether the pro-apoptotic and anti-metastatic effects are mediated by the cav-1/AKT pathway, LY294002, a PI3K inhibitor, was used to knockdown the expression of AKT. We found that LY294002 could markedly increase the baicalein-induced anti-cancer effect. In addition, it has been reported that AKT exerts its biological effect by phosphorylating its downstream substrates, including mTOR. Phosphorylated mTOR can promote the proliferation, motility, and survival of cancer cells [41]. The results indicated that baicalein treatment also inhibits the mTOR phosphorylation and AKT activity. Thus, these data suggest that baicalein may inhibit the cav-1/AKT/mTOR pathway to induce its anti-cancer effect.

Additionally, Akt is reported to promote cell survival through the direct regulation of Bcl-2 family members [42, 43]. Our data also demonstrated that the inhibition of AKT activity can enhance the upregulation of Bax expression and downregulation of Bcl-2 expression induced by baicalein, suggesting that AKT may be at the upstream of the baicalein-induced apoptosis of prostate cancer cells.

Taken altogether, our study demonstrates that baicalein markedly induces apoptosis and inhibits metastasis of androgen-independent prostate cancer cells. Mechanically, we find that cav-1/AKT/mTOR pathways account for the anti-tumor effects of baicalein. All these results imply that baicalein may be a promising therapeutic drug for androgen-independent prostate cancer patients.
